# Professorship of Music in the Normal Schools

**Published:** 1855-03

**Authors:** 


Professorship of Music in the Normal Schools.—Mr. Randall, City Superintendent of Schools, has warmly recommended to the Board of Education the appointment of a Professor of Music in the Normal Schools. His arguments are:

The introduction of music, both vocal and instrumental, in our public schools, has become general, and has received the unqualified sanction of the most enlightened and practical friends of education. Through its agency, to a very great extent, a complete revolution has been effected in the order, harmony, and discipline of these institutions; and its elevating and beneficial effects have have been sensibly perceived in the moral influence exerted upon the minds of the pupils. The time has arrived when this noble and useful art should be scientifically taught in our schools, by the ablest and most accomplished professors; when every teacher should obtain a thorough and familiar knowledge of its principles, with the ability to communicate that knowledge practically and effectively to his pupils; and when in all the higher and more advanced departments of the schools its cultivation should be systematically pursued. The establishment of a Professorship in the Normal Schools, where the science could be taught methodically and uniformly, could not fail, in the judgment of the undersigned, essentially to promote the best interests of education in this respect. It is not perceived, however, that any substantial advantage could accrue from the extension of this plan to the Evening Schools. The limited time at the disposal of this class of pupils will not admit of the introduction of any branches of science not of immediate and practical utility.

